# The Impact of Parental Behaviors on Children’s Lifestyle, Dietary Habits, Screen Time, Sleep Patterns, Mental Health, and BMI: A Scoping Review

**DOI:** 10.3390/children12020203

**Published:** 2025-02-08

**Authors:** Cátia Maia, Diogo Braz, Helder Miguel Fernandes, Hugo Sarmento, Aristides M. Machado-Rodrigues

**Affiliations:** 1 University of Coimbra, Faculty of Sport Sciences and Physical Education, 3040-248 Coimbra, Portugal; uc2017263629@student.uc.pt (C.M.); uc2018280301@student.uc.pt (D.B.); hugo.sarmento@uc.pt (H.S.); 2 School of Education, Communication and Sports, Polytechnic Institute of Guarda, 6300-559 Guarda, Portugal; hmfernandes@ipg.pt; 3 Sport Physical Activity and Health Research & INnovationCenTer (SPRINT), 6300-559 Guarda, Portugal; 4 University of Coimbra, Interdisciplinary Center for the Study of Human Performance (CIPER-UC), 3040-248 Coimbra, Portugal; 5 Research Centre for Anthropology and Health, University of Coimbra, 3000-456 Coimbra, Portugal

**Keywords:** lifestyles, mental health, overweight, obesity, youths, families

## Abstract

Background and Objectives: Childhood obesity and being overweight are influenced by the family environment, diet, sleep, and mental health, with parents playing a key role in shaping behaviors through routines and practices. Healthy parental habits can encourage positive outcomes, while poor routines and stress often lead to unhealthy weight gain. This study analyzed the impact of parental behaviors on children’s lifestyles and habits, as well as the trend and intensity of the effect of these behaviors on different age groups. Methods: A systematic review of 1504 articles from Web of Science, PubMed, Scopus, and APA PsycNet (as of 22 July 2024) included studies on parents and children aged 4–18 years, focusing on physical activity, sleep, screen time, nutrition, and mental health. Twenty-six studies were analyzed, including 19 cross-sectional and 7 longitudinal studies. The outcomes included physical activity, sedentary behaviors, eating and sleeping habits, mental health, and BMI. Bias was assessed using JBI tools according to the GRADE framework and Newcastle-Ottawa Quality Assessment. Results: The studies involved 89,545 youths and 13,856 parents. The key findings revealed associations between parental physical activity, sleep, dietary habits, mental health, screen time, and their children’s BMIs. Parenting styles significantly influence children’s behaviors. This review highlights the crucial influence of parenting styles and behaviors on children’s physical activity, diet, sleep, and mental health, emphasizing the link between family dynamics and childhood obesity. The findings stress the importance of targeting parental habits in interventions focused on healthy routines and stress management. Longitudinal studies are needed to determine causality, while research involving diverse populations is essential to enhance the applicability of these findings.

## 1. Introduction

The rising issue of being overweight and obese among youths has become a significant area of research aimed at developing effective prevention and intervention strategies. Addressing the increasing prevalence of these conditions is complex, as it requires more than simply promoting dietary changes and encouraging daily moderate-to-vigorous physical activity. By the late 20th century, researchers began to examine systemic family dynamics related to childhood and adolescent obesity, emphasizing parenting styles and family interactions [1]. Various factors, including the family environment, lifestyle choices, dietary habits, sleep patterns, and mental health, significantly impact the increasing rates of obese and overweight youths [2,3,4]. Family systems theory posits that families function within complex networks where many interactions co-occur [5]. These interactions are reciprocal, meaning that they mutually influence each other and shape the dynamics within a family. Family dynamics and parental feeding styles are essential in shaping children’s development. Parents play a crucial role by creating the food environment and influencing key aspects of their children’s diet, sleep, and physical activity. This shows a strong correlation between the body mass index (BMI) of parents and that of their children [6,7], with parental dietary habits being particularly influential [6,8]. Research indicates that maternal obesity or being overweight is strongly associated with a higher likelihood of being overweight or obese in childhood, as well as the continuation of excess weight [9]. Specifically, children of overweight or obese parents face a 12% increased risk of becoming obese themselves. Other researchers suggest that the likelihood of a child being overweight or obese increases by 1.62 times when one parent is overweight or obese. If both parents are overweight or obese, then this probability increases three times [10].

Additionally, socioeconomic status (SES), which can be measured through parental education levels, indicates that parents with higher levels of education are more likely to make informed health-related choices. They tend to adopt healthier lifestyles and be positive role models for their children [11]. Parents with lower education levels frequently reside in low-income neighborhoods, which tend to have environments that contribute to obesity [12]. Additionally, family dynamics play a significant role in childhood obesity. Siblings can encourage each other to participate in more physical activities and outdoor play [11]. In contrast, children without siblings may be more inclined to engage in sedentary behaviors, like watching TV [13].

Evidence suggests that the eating behaviors of young people are closely linked to their parents’ dietary practices [8,14,15]. Parental emotional eating is often associated with the emotional malnourishment of their children [14]. Costa and Oliveira [16] concluded that less healthy eating habits are correlated with youth behavioral issues and parental stress. This relationship may influence how parents perceive feeding challenges, impacting the overall quality of their children’s diets. According to family systems theory [5], an authoritative feeding style is expected to promote healthier dietary habits and enhance a child’s ability to self-regulate their eating behaviors. In contrast, an authoritarian feeding style will likely result in more rigid feeding practices and a diminished ability to recognize and respond to hunger and fullness cues [5,11,17]. Therefore, parental feeding styles can significantly affect the dietary quality of youths [11,14,15]. A more permissive feeding style, often stemming from parental stress, anxiety, and depression [18], tends to result in fewer feeding challenges for children and is associated with higher body mass index (BMI) levels.

In addition, controlling and persuasive feeding practices are associated with symptoms of anxiety and depression. These practices not only increase the risk of being overweight and obesity but may also promote emotional eating patterns, leading to excessive or insufficient food intake [11,18,19,20]. Specifically, sleeping for less than 7 h increases the risk of being overweight and obesity [20]. Kanellopoulou and Notara [19] further reinforced this idea by highlighting a negative association between average sleep duration and the likelihood of being overweight or obese.

Healthy lifestyles among parents can help reduce childhood obesity. When parents embrace healthier habits, the risk of being overweight for youths is lower [6,21]. In this context, physical activity plays a crucial mediating role, as the level of physical activity parents engage in significantly influences how much physical activity their children undertake [14,22]. There is a positive correlation between parents’ physical activity and their mental health, as well as a similar positive relationship between the physical activity levels of youths and their mental health. Additionally, the quality of family relationships is vital for mental health, as it is closely linked to the prevalence of depression and anxiety disorders [23,24]. Additionally, family conflicts are strongly associated with an increase in mental health issues, underscoring the critical impact of family cohesion on psychological well-being [24].

Given these connections, this review aims to consolidate scientific evidence regarding the nature of associations between parents and their children. Specifically, it seeks to explore whether there are notable differences in the intensity and trends of these associations over time. Furthermore, this review analyzes how parental habits and behaviors influence their children’s lives, including lifestyle choices, eating habits, screen time, sleep patterns, mental health, and body mass index (BMI). Finally, an important goal is to assess whether the evidence gathered in recent decades remains consistent and relevant in understanding these dynamics today.

## 2. Materials and Methods

### 2.1. Protocol and Registration

This review was prepared while following the PRISMA Extension for Scoping Reviews (PRISMA ScR) (Supplementary Table S1). The protocol for this review is registered with the Open Science Framework (OSF) at the following link: https://doi.org/10.17605/OSF.IO/NRK7T.

### 2.2. Eligibility Criteria

This review adhered to the population, interventions, comparisons, outcomes, and study design (PICOS) framework [25] to identify the essential concepts of the studies related to the research question in advance and to streamline the search process.

This review included studies published in English, encompassing qualitative, quantitative, longitudinal, and cross-sectional research which did not involve interventions. It specifically examined the relationships between parental characteristics and behaviors—such as lifestyle, sleep habits, physical activity, and mental health—and those of their children. The analysis focused on families with children and adolescents aged 4–18, exploring the association between parental behaviors and children’s lifestyle outcomes. The review considered interventions related to parental eating patterns, physical activity levels, sleep habits, and parenting styles. Comparisons assessed variations in the children’s physical activity, body mass indexes (BMIs), sleep quality and duration, screen time, dietary habits, and mental health. Only peer-reviewed studies with longitudinal designs were included, while non-randomized experimental studies and the gray literature were excluded. The outcomes highlighted the significant impact of family dynamics on children’s well-being.

### 2.3. Literature Search and Study Selection

A comprehensive review was conducted on 22 July 2024 across four research databases—Web of Science, PubMed, Scopus, and APA PsycNet—using common keywords and the Boolean operators AND and OR based on the following search equation: [“Adolescent*” OR “Teenager*” OR “teen” OR “family”] AND [“Adiposity” OR “Normal weight” OR “overweight” OR “obesity” OR “BMI”] AND [“lifestyle*” OR “physical activity” OR “sedentary lifestyle” OR “sleep habit*” OR “nutritional habit*” OR “family environment*”] AND [“mental health” OR “anxiety” OR “stress” OR “depress*” OR “well-being” OR “body satisfaction”]. Table 1 outlines the search strategy used for each database. Original research studies on children, adolescents, and families were included. No restriction was placed on the publication year, although the synthesis was limited to studies in English. The articles were imported into EndNote X9.3.3 software (Clarivate Analytics, Philadelphia, PA, USA), where the gray literature was excluded, and duplicates were automatically and manually removed.

### 2.4. Inclusion and Exclusion Criteria

The inclusion criteria were as follows: (1) articles written in English; (2) nuclear, co-parenting, and single-parent families; (3) children aged between 4 and 18 years; (4) analysis of the habits and lifestyles of both parents and children; and (5) analysis of the sleep habits, physical activity, and mental health of both parents and children.

The exclusion criteria were as follows: (1) children residing in social institutions; (2) children younger than 4 years or older than 18 years; (3) families with chronic diseases (e.g., diabetes or hypertension); (4) families with psychological or mental illnesses; (5) surgical interventions; and (6) studies implementing exercise training, diet plans, or intervention programs. Interventional studies were excluded because their focus is on evaluating intervention effectiveness, while this review aims to examine natural risk factors and associations without manipulating variables. This approach emphasizes real-world relationships between factors rather than testing interventions.

### 2.5. Data Extraction

Two authors (C.M. and D.B.) conducted the extraction of original bibliographic articles. The two authors conducted the search and selection independently using the following criteria: the characteristics of the target population, age of the target population, type of study, and outcome. When necessary, a third and fourth author (A.M.R. and H.F.) assisted in including or excluding articles.

### 2.6. Data Items, Synthesis, and Charting

Once the final selection of articles was performed, data from eligible articles were extracted and organized in a Microsoft Excel file. The data categories included author(s), year of publication, article title, type of study (e.g., cross-sectional, cohort, or longitudinal), objectives and purpose, population characteristics (sample size and average age), BMI, physical activity measures, sleep measures, screen time measures, mental health, family characteristics, nutritional habits, and primary study results. In this process, the articles were grouped according to the variables under study. For this scoping analysis, the findings for each research question were presented based on the articles which provided evidence of a positive, negative, or no association between the habits of young people and their families’ habits.

### 2.7. Risk of Bias and Study Quality Assessment

The quality of the included cross-sectional and cohort studies was assessed using critical appraisal tools from Joanna Briggs Institute (JBI) [26], which comply with the recommendations of the Grading of Recommendations Assessment, Development, and Evaluation (GRADE) framework, to analyze the quality of primary research and the risk of bias. These tools consist of an 8 item checklist for analytical cross-sectional studies and a 10 item checklist for qualitative studies. Each checklist offers four response options for every question: “yes = 1”, “no = 0”, “unclear = 0”, and “not applicable = not scored”. These responses guide the assessment of the methodological quality of the studies. For the 8 item checklist and 10 item checklist, a percentage of 0–33% indicates low quality, a rate of 34–66% indicates moderate quality, and a rate of 67% or more indicates high quality [27,28].

The quality of the included longitudinal and cohort studies was evaluated using the Newcastle-Ottawa Quality Assessment Form [29,30]. The scale uses a scoring system based on three main domains: selection, which evaluates how participants were recruited and whether the initial cohort was representative of the target population; comparability, which assesses the study’s ability to control for confounding factors, ensuring that the cohorts were comparable regarding the most relevant variables; and outcome, which examines the quality and consistency in the assessment of outcomes as well as the adequacy of the follow-up period. The Newcastle-Ottawa Scale (NOS) [29,30] would be converted to AHRQ quality standards, classifying studies as good, fair, or poor. Good quality requires 3–4 stars for selection, 1–2 for comparability, and 2–3 for outcome or exposure. Fair quality needs 2 stars for selection, 1–2 for comparability, and 2–3 for outcome or exposure. Studies scoring 0–1 in selection, 0 in comparability, or 0–1 in outcome or exposure were rated as poor quality. This ensured standardized study evaluation.

Among all of the above-mentioned tools, the NOS is the most used tool currently, and it can also be modified based on a special subject [31].

## 3. Results

### 3.1. Selection and Description of Studies

The literature search was conducted across the Web of Science, PubMed, Scopus, and APA PsycNet databases, producing 1504 articles. An additional seven articles were sourced from other research databases. Following the PRISMA guidelines, the search and selection process was conducted independently by C.M. and D.B. Duplicate articles were removed using EndNote X9.3.3 software (Clarivate Analytics, Philadelphia, PA, USA), reducing the total number of articles to 1093. After the titles and abstracts were reviewed, 1049 articles were excluded. Thirty-seven articles were assessed for eligibility based on the inclusion and exclusion criteria, and 18 met the eligibility criteria. Additionally, eight studies were identified via other methods [32,33,34,35,36,37].

Consequently, 25 studies were included in this review.

**Figure 1:**
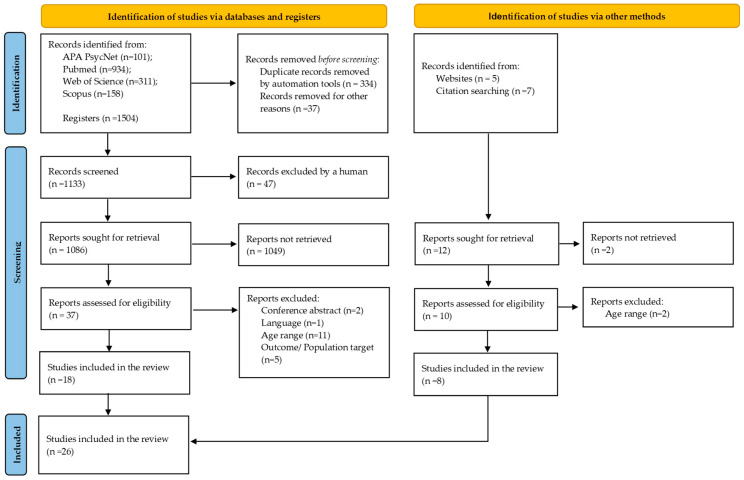
PRISMA diagram for the identification, screening, eligibility, and inclusion of studies.

Of the 19 excluded articles, 11 included an inappropriate age range, 5 presented decontextualized outcomes, 2 were conference abstracts, and 1 was a Mendelian randomization study [38]. Nineteen of the included studies were cross-sectional, and six were longitudinal, with participants aged between 4 and 18 years. One of the included cross-sectional studies was a qualitative study [39]. The publication years ranged from 2004 to 2024. Among the included articles, only 2008, 2010, 2014, 2018, and 2019 had a single article each. No articles from 2016 or 2021 were included. Two articles from 2004, 2015, 2022, and 2024, three from 2017, five from 2023, and five from 2020 were analyzed. Figure 1 shows the selection process of the included studies.

Among the 25 articles, 13 assessed physical activity and sedentary behavior [6,33,35,37,39,40,41,42,43,44,45,46,47], 4 evaluated sleep habits and quality [32,34,48,49], and 5 measured screen time [32,40,41,43,50]. Mental health was examined in 13 articles [37,42,43,44,45,46,47,51,52,53,54,55,56], whereas the family environment was analyzed in all articles except one [6]. Fifteen studies addressed dietary habits [6,14,35,37,41,43,44,46,47,50,51,52,53,54,56].

The BMI was calculated through weight measurement using calibrated electronic scales and self-reported data, and height measurements were obtained using a stadiometer and self-reported data. Physical activity and sedentary behavior data were collected using self-reporting questionnaires [6,35,36,37,39,40,41,43,44,45,46,47] and accelerometers [33,42]. Sleep habits and quality of sleep, screen time, mental health, family environment and characteristics, and nutritional and eating habits were measured using questionnaires.

The Joanna Briggs Institute’s (JBI) critical appraisal tools [26] were applied, and the results showed moderate-to-high quality, ranging from 62.5% to 87.5%. Regarding the Newcastle-Ottawa Quality Assessment Form (NOS) [29,30], two articles were of “fair” quality, and five were of “good” quality [46,47,50,51]. The results of Joanna Briggs Institute’s (JBI) critical appraisal tools and the Newcastle-Ottawa Quality Assessment Form (NOS) are in Table 2, Table 3 and Table 4.

### 3.2. Main Findings

Table 5 demonstrates an association between parental lifestyles and the unhealthy behaviors adopted by their children. Socioeconomic factors are linked to obesity, with evidence indicating that low-income families are more likely to have obese children [36,43,51,55,56]. Youth mental health is closely related to obesity. Research has shown that the risk of obesity is four times higher among youths who exhibit anxiety traits [51] and depressive symptoms [47,53,55]. Additionally, parental mental health significantly impacts their children’s well-being, habits, and weight [44,45,47,52,53]. Studies have indicated that better mental health in mothers and fathers is associated with a lower likelihood of their children being overweight or obese [43,45,47,56].

Stress is a critical factor in mental health. When parental stress levels are high, they can negatively affect children’s physical activity and overall well-being [42,45], despite parents recognizing the importance of physical activity in family life [39]. Only a small percentage of youths are aware of and meet the recommended daily physical activity levels [35], with boys being more likely to meet these guidelines [35,40]. However, boys also exhibit lower levels of physical activity and more television viewing, particularly among those who are overweight compared with their healthy-weight peers [54]. Parental habits, behaviors, and lifestyles can influence those of their children [46,47]. Therefore, increased physical activity among mothers and fathers and participation in family physical activities can help foster active and consistent exercise habits in youths [33,35,36,39,40,41,42,45,47,50]. This, in turn, can address sedentary behaviors, especially excessive screen time, which heightens the risk of obesity [33,41,42]. Such sedentary behaviors can increase junk food consumption and contribute to unhealthy lifestyles [41]. Improving the quality of the family meal environment may also enhance overall physical fitness and reduce daily soft drink consumption [46]. Furthermore, dietary and sleep habits are crucial factors related to obesity. Families with unstable and unhealthy dietary patterns [14,37,41,44,52] tend to negatively affect their children’s well-being and sleeping habits [32,34,48,49], which can subsequently impact mental health and increase the risk of being overweight or obese.

## 4. Discussion

This review synthesized findings from 22 studies examining the relationships between lifestyle factors, sleep patterns, dietary habits, mental health, and parenting styles in parents and youths of ages 4–18. The total sample included 59,360 youths and 13,428 parents.

Only two studies [37,48] received a moderate quality rating; therefore, their results did not significantly impact the overall findings. However, these studies were less robust and had a higher risk of bias. Their inclusion and analysis were carried out carefully, considering factors such as weighting of evidence, subgroup analysis, and overall confidence in evidence to acknowledge their potential impact on the results.

The analysis revealed significant correlations between the lifestyles, habits, and behaviors of parents and their children, notably affecting their overall well-being and body mass index (BMI) scores. Key aspects of the family environment, such as socioeconomic status (SES), dietary practices, and parenting styles, emerged as crucial factors influencing the development of unhealthy behaviors. These factors include eating habits, sleep patterns, screen time, and physical activity.

### 4.1. Family Environment, Characteristics, and BMI

Socioeconomic status significantly influences the BMIs of youths [35,46,47,50,51]. However, research has shown that various family-related factors contribute to youth BMIs [57,58,59]. Additionally, the family climate and environment can impact academic performance and general success or failure in school [57].

For example, permissive parenting styles or indulgent parenting styles, whether from the mother or father [58,60], are commonly linked to higher BMI values. This happens because permissive or indulgent parenting allows youths the freedom to adopt unhealthy behaviors, particularly regarding their eating habits. These parenting approaches may lead to the easier acceptance of processed and convenient food options to avoid conflicts or difficulties around mealtimes [59,60].

Similarly, a lack of parental presence or supervision can lead to increased consumption of convenient yet unhealthy foods without parental control, often with little awareness of the benefits or harms of such dietary choices. A dysfunctional family environment may cause psychological changes in youths and contribute to disordered eating behaviors, like loss of control and overeating, stemming from a lack of emotional expression from parents [52,58]. Fostering a supportive family environment can lead to significant improvements despite these challenges. Authoritative parenting, shared meals, open communication, and consistent guidance encourage healthier habits, enhance emotional well-being, and strengthen family bonds, ultimately promoting positive long-term outcomes.

### 4.2. Physical Activity and Sedentary Behavior

Recent research on the relationship between parents’ body mass indexes (BMI) and their children’s BMIs indicated that children with overweight or obese parents are significantly more likely to be overweight or obese themselves [6,7,61,62,63,64,65]. There is also a strong correlation between parents’ physical activity levels and those of their children [22,33,35,39,45,47], as parents often serve as role models. Studies have shown that when parents engage in less physical activity or lack awareness of its benefits, their children tend to mirror these behaviors, leading to reduced physical activity and increased BMIs [66,67].

The importance of physical activity for youth well-being is underscored by recent findings reiterating earlier research [35]. However, most young people fail to meet the recommended physical activity guidelines [68,69]. For instance, a study investigating the link between sugar-sweetened beverage (SSB) consumption and levels of moderate and vigorous physical activity among youths—especially those who are overweight or obese—found that higher SSB intake, along with inadequate physical activity, increased the risk of being overweight or obese [70,71]. Research has shown a direct link between low physical activity levels and an increased risk of obesity. SES also affects physical activity habits, as communities with higher SESs often provide better resources and infrastructure which promote healthy behaviors. These areas generally have more amenities and facilities encouraging active lifestyles [72,73].

Additionally, regular physical activity delivers numerous health benefits, including lower levels of specific hormones which can affect food intake and contribute to more effective weight management [74]. Employing family-based approaches can promote healthier lifestyles, as parental habits significantly impact children’s body mass indexes (BMIs) and activity levels. Encouraging parents to engage in regular physical activity and adopt healthier dietary choices can create a supportive environment for positive change. Collaborative programs in schools and communities can focus on family-centered routines, reducing the risk of obesity while improving the overall well-being of youths.

### 4.3. Nutritional Habits and Eating Habits

The findings indicate a significant correlation between parents’ eating habits, children’s nutritional statuses [37], and their eating behaviors. This relationship is influenced by factors such as nutritional literacy and the pressure parents exert on their children to eat, which have been shown to impact youths’ eating behaviors [44]. Research corroborating these findings [18,49,59,75] highlights that certain parenting styles probably contribute to increased consumption of unhealthy foods, which is associated with higher BMIs among youths [59].

Moreover, parents’ perceptions of their children’s weight may be crucial in shaping their feeding practices, significantly influencing their children’s body image and the stigma associated with weight [15]. Recent studies further underscored the connection between parents’ mental health and the dietary quality of their children [18]. These studies revealed that poorer mental health among parents often leads to more controlling and coercive feeding strategies, which can adversely affect youths’ nutritional well-being. Such feeding practices may disrupt youths’ ability to self-regulate their food intake, potentially increasing the risk of obesity in later stages of life [76].

### 4.4. Mental Health

Parents’ mental health plays a significant role in their children’s well-being, particularly concerning physical activity levels. Research has demonstrated a negative correlation between parental stress and youth’s engagement in physical activities. Conversely, effective family communication has been shown to influence physical activity levels [45].

Recent studies have further emphasized the connection between parents’ mental health and youth’s tendencies toward emotional eating [18,75]. Specifically, youth whose parents experience poorer mental health may exhibit higher levels of emotional eating, indicating a link between the emotional states of parents and their children’s eating behaviors.

Additionally, youths’ perceptions of body weight and experiences of food restriction significantly impact their mental health. Such perceptions can foster body stigma, leading to detrimental changes in dietary habits [15]. Furthermore, a higher body mass index (BMI) has been correlated with mild depressive symptoms as well as slight alterations in anxiety levels and attention-deficit hyperactivity disorder (ADHD) among youths [38]. Notably, this research suggests that a mother’s BMI may also have a direct influence on her child’s depressive symptoms, underscoring the interconnectedness of familial health dynamics [38]. Another factor which significantly influences mental health is screen time, particularly the time spent on online gaming. While there are some benefits to electronic gaming, such as fostering creativity and improving cognitive functioning [77], the long-term negative impacts on mental health if gaming is overemphasized [68] and on family relationships as a means of escaping real-life situations are evident [78]. Therefore, understanding the dual nature of gaming’s impact on mental health is crucial to promoting healthier engagement practices. Promoting open family communication and mental health support for parents can reduce stress and positively impact children’s behaviors, including physical activity and emotional eating. Encouraging body positivity and family-based healthy habits can further improve mental and physical health outcomes, fostering a supportive environment for overall well-being.

### 4.5. Screen Time

Screen time remains a frequent topic of discussion among families, considering both benefits and disadvantages. Parents tend to implement strategies and behaviors to reduce screen time more than promote physical activity levels [79], recognizing the adverse effects that screens can have [80,81]. The significant consequences of excessive screen time include impacts on youths’ healthy development [82], overall well-being [83,84], relationships with their parents [80,81,85], and social-emotional growth [86,87,88].

While some studies have highlighted the positive aspects of screens [77], particularly in educational contexts such as reading [89], excessive screen use has detrimental effects on cognitive development, negatively influencing academic performance [89,90]. Language development is notably impacted, especially among younger children, as this limits their interactions with peers, adults, and parents [85,88].

Studies have shown that children aged 8–17 spend an average of 1.5–2 h playing video games daily [91]. In this context, active parental monitoring, including setting rules and limits on screen time [80,81] and engaging in open discussions about game content, is essential for fostering healthy gaming behaviors and mitigating potential risks [92]. A lack of supervision may lead to problematic gaming habits, where adolescents turn to video games to cope with negative emotions such as stress or frustration [92,93].

The visual content consumed can trigger behavioral changes, potentially contributing to ADHD [94]. Furthermore, screen time has been linked to obesity, anxiety, depression [55], and sleep disturbances [87,95]. Socioeconomic status is a variable which influences screen time exposure and duration. Studies have indicated that children from families with high socioeconomic statuses (SESs) spend significantly less time with screens compared with those from middle- and low-SES groups [73].

### 4.6. Quality and Sleep Habits

The duration and quality of sleep are influenced by several factors, especially screen time [80,95]. Parents’ habits and behaviors also play a key role in determining sleep quality, as they establish routines which impact youths’ overall well-being. One consequence of poor sleep habits is obesity, with research indicating that a 2 h delay in bedtime at the age of 11 predicted a 0.6 cm increase in waist circumference after a 2.5 year follow-up period [96]. Additionally, inconsistent sleep patterns can cause hormonal imbalances which affect appetite and energy levels, increasing the risk of obesity [97,98].

Furthermore, irregular sleep can lead to low energy, poor concentration, and mood swings. In youths aged 4–12 years, the most common sleep issues are falling asleep and staying asleep [99], with anxiety and disrupted routines being the main contributors. The family environment might significantly influence sleep quality [100,101,102,103], particularly the relationship between parents and children [104,105]. Spending time with family can help reduce anxiety and improve one’s well-being, leading to better sleep quality. This relationship depends on family dynamics, especially parental relationships, and factors such as the child’s gender, alcohol consumption, and parental education levels. The likelihood of a youth getting adequate sleep increases with the parents’ level of education [103].

The analysis highlights the positive impact of a supportive family environment on young people’s BMIs, emphasizing the importance of healthy parental behaviors and mental well-being. Socioeconomic status is a factor which also influences family lifestyles and is associated with the prevalence of obesity and being overweight [106], with increases being observed particularly in socioeconomically disadvantaged contexts [72]. The environment in which children grow up can significantly impact their dietary choices and levels of physical activity.

Improving parenting styles, promoting physical activity, managing screen time, and ensuring adequate sleep can significantly enhance youths’ well-being and reduce obesity risks. Parental mental health fosters emotional eating habits and encourages physical activity. By prioritizing family support and healthy habits, we can create an environment which promotes positive outcomes for children and adolescents.

### 4.7. Study Strengths and Limitations

This review has several strengths, like the well-defined inclusion and exclusion criteria and focusing on specific age groups (4–18 years) and family types while excluding studies with confounding variables, ensuring the review’s relevance. It includes a diverse population, covering various family compositions and parental behaviors and providing a holistic view of the family environment’s impact on children. Including qualitative and quantitative non-interventional studies, it captures a broad spectrum of observational data, offering insights into naturalistic family dynamics. A systematic approach for data extraction, multiple reviewers to minimize bias, and thorough assessments of study quality and risk of bias enhance this review’s robustness.

Overall, this study provides a comprehensive and methodologically sound review of the association between parental behaviors and many child outcomes, highlighting significant associations and potential areas for intervention. However, limitations such as language restrictions, the predominance of cross-sectional studies, limited consideration of cultural factors, and biases should be considered. Including studies with self-reported data may raise concerns about the exact accuracy of some findings; however, these data collection tools are commonly employed in studies investigating multiple variables with large sample sizes. Future research could benefit from a broader range of studies and culturally diverse contexts as well as longitudinal and interventional models to strengthen causal inferences.

## 5. Conclusions

An active lifestyle is crucial for the well-being of children and adolescents. Excessive screen time and inconsistent sleep routines can harm both physical and mental health, fostering unhealthy habits. Balanced parenting plays a key role in shaping eating behaviors and self-regulation, reducing the risk of obesity and related issues, such as eating disorders.

This review highlights the connection between parental behaviors and children’s BMIs, eating habits, physical activity, and sleep patterns. However, several aspects remain underexplored, such as the long-term effects of parental influence as children grow into adolescence and how cultural, socioeconomic, and environmental factors affect these behaviors. Although the evidence did not definitively show whether the intensity of these associations changes with age, it did emphasize the significant role parental behaviors play in shaping the lifestyles of young people, in line with previous data.

Practical strategies involving both parents and children are essential to fill these gaps. Future interventions should focus on educating parents on managing screen time, promoting physical activity, and fostering healthy eating habits at home. Additionally, offering mental health support to parents can reduce stress and improve family dynamics and children’s behaviors. Schools and community programs should work together to develop family-focused initiatives which reinforce these positive habits, helping to reduce the risk of obesity and improve youth well-being.

## Figures and Tables

**Table 1:** Search strategy.

Database	Type of Research	Search Strategy	N. Articles
Web of Science	Topic and Title	Adolescent* OR Teenager* OR teen OR family (Topic) AND Adiposity OR “Normal weight” OR overweight OR obesity OR BMI (Topic) AND lifestyle* OR “physical activity” OR “sedentary lifestyle” OR “sleep habit*” OR “nutritional habit* “ OR “family environment*” (Title) AND “mental health” OR anxiety OR stress OR depress* OR well-being OR “body satisfaction” (Title).	311
Pubmed	Title or Abstract	(adolescent[Title/Abstract] OR teenager*[Title/Abstract] OR teen[Title/Abstract] OR family[Title/Abstract]) AND (adiposity[Title/Abstract] OR “Normal weight”[Title/Abstract] OR overweight[Title/Abstract] OR obesity[Title/Abstract] OR bmi[Title/Abstract]) AND (lifestyle[Title/Abstract] OR “physical activity”[Title/Abstract] OR “sedentary lifestyle”[Title/Abstract] OR “sleep habits”[Title/Abstract] OR “nutritional habits”[Title/Abstract] OR “family environment”[Title/Abstract]) AND (“mental health”[Title/Abstract] OR anxiety[Title/Abstract] OR stress[Title/Abstract] OR depress[Title/Abstract] OR well-being[Title/Abstract] OR “body satisfaction”[Title/Abstract]).	934
Scopus	Title, Abstract, or Keywords	(TITLE-ABS-KEY (adolescent* OR teenager* OR teen OR family) AND TITLE-ABS-KEY (adiposity OR “Normal weight” OR overweight OR obesity OR bmi) AND TITLE ( lifestyle* OR “physical activity” OR “sedentary lifestyle” OR “sleep habit*” OR “nutritional habit*“ OR “family environment*”) AND TITLE (“mental health” OR anxiety OR stress OR depress* OR well-being OR “body satisfaction”)) AND PUBYEAR > 1979 AND PUBYEAR < 2025 AND (LIMIT-TO (DOCTYPE, “ar”)) AND (LIMIT-TO (LANGUAGE, “English”)).	158
APA PsycNet	Abstract and Title	((adolescent* or teenager* or teen or family) and (adiposity or “Normal weight” or overweight or obesity or bmi)). ab. and (lifestyle* or “physical activity” or “sedentary lifestyle” or “sleep habit*” or “nutritional habit* “ or “family environment*”).ti. and (“mental health” or anxiety or stress or depress* or well-being or “body satisfaction”).ti.	101

**Table 2:** Quality analysis of cross-sectional studies.

	JBI Critical Appraisal Checklist for Analytical Cross-Sectional Studies									
	1.	2.	3.	4.	5.	6.	7.	8.	Total	Quality
Ari et al. [32]	Yes	Unc	Yes	Yes	Unc	Yes	Yes	Yes	75%	High quality
Bracale et al. [41]	Yes	Unc	No	Yes	Yes	Yes	Yes	Yes	75%	High quality
Brouwer et al. [33]	Yes	Unc	Yes	Yes	Yes	Yes	Yes	Yes	87.5%	High quality
Cadogan et al. [40]	Yes	Yes	Yes	Unc	Yes	Yes	Yes	Yes	87.5%	High quality
Chehri et al. [34]	Yes	Unc	Yes	Yes	Yes	Yes	Yes	Yes	87.5%	High quality
Dozier et al. [35]	Yes	Yes	Yes	Yes	Unc	Yes	Yes	Yes	87.5%	High quality
Eley et al. [53]	Yes	No	Yes	Yes	Yes	Yes	Yes	Yes	87.5%	High quality
Foster et al. [43]	Yes	Yes	Unc	Yes	Yes	Yes	Yes	Yes	87.5%	High quality
Kobayashi et al. [45]	Yes	Yes	Yes	Unc	Unc	Yes	Yes	Yes	75%	High quality
Maher et al. [42]	Yes	Unc	Yes	Unc	Yes	Yes	Yes	Yes	75%	High quality
Makinen et al. [54]	Yes	Yes	Yes	Yes	Unc	Yes	Yes	Yes	87.5%	High quality
Martins et al. [48]	Yes	Yes	Unc	Yes	No	No	Yes	No	62.5%	Moderate quality
Qiu et al. [14]	Yes	Yes	Yes	Unc	Unc	Yes	Yes	Yes	75%	High quality
Sepúlveda et al. [52]	Yes	Yes	Yes	Yes	Unc	Yes	Yes	Yes	87.5%	High quality
Tommasi et al. [37]	Yes	Yes	Unc	Unc	Unc	Yes	Yes	Yes	62.5%	Moderate quality
Tsai et al. [49]	Yes	Yes	Yes	No	No	Yes	Yes	Yes	75%	High quality
Zhu et al. [44]	Yes	Yes	Unc	NA	Yes	Yes	Yes	Yes	75%	High quality

**Table 3:** Quality analysis of qualitative research.

	JBI Critical Appraisal Checklist for Qualitative Research											
	1.	2.	3.	4.	5.	6.	7.	8.	9.	10.	Total	Quality
Thompson et al. [39]	Yes	Yes	Yes	Yes	Yes	No	Unc	Yes	Yes	Yes	80%	High quality

**Table 4:** Quality analysis of longitudinal and cohort studies.

	Newcastle-Ottawa Quality Assessment Form (NOS) for Cohort Studies								
	Selection				Comparability	Outcome			
	1.	2.	3.	4.	5.	6.	7.	8.	Quality
Benny et al. [55]	X	X			XX		X	X	Fair
Harbec et al. [46]	X	X		X	X	X	X	X	Good
Hoehne et al. [36]	X	X	X	X	X	X	X	X	Good
Monteiro et al. [51]	X	X	X	X	XX		X	X	Good
Neumark-Sztainer et al. [50]	X	X	X	X	X		X	X	Good
Peng et al. [56]	X	X		X	XX		X	X	Good
Wang et al. [47]		X		X	X		X	X	Fair
Webber-Richey et al. [6]	X		X	X	XX		X	X	Good

Good quality = 3 or 4 stars in the selection domain, 1 or 2 stars in the comparability domain, and 2 or 3 stars in the outcome or exposure domain; fair quality = 2 stars in the selection domain, 1 or 2 stars in the comparability domain, and 2 or 3 stars in the outcome or exposure domain.

**Table 5:** Results.

Authors, Year, Study Design		Age (yrs)	*N* and Statistical Analysis	Sleep Measure		Screen Time Measure	Family Environment and Characteristics		Main Findings		
Ari et al., 2023 [32]. Cross-sectional		6–15	*N* = 225 households Descriptive statistics, non-parametric tests, stepwise regression, path analysis	Time in bed (TIB) was calculated by investigating students’ habitual bedtimes and wake-up times and their parents’ bedtimes and wake-up times on weekdays.		Screen time was assessed by reporting children’s average daily usage times (excluding study usage) over the past week, including weekends. Screen time was recorded in 30 min intervals, ranging from 0 to 300 min.	The background information was collected from the questionnaire survey. Children’s Strengths and Difficulties Questionnaire (SDQ) was administered to parents to assess each child’s difficulties.		The significant challenges identified by the SDQ and shorter sleep durations were linked to increased screen time among elementary and junior high school students. A negative relationship was found between parental bedtime and children’s sleep duration (β = 0.325, *p* < 0.001). However, no significant direct or overall effect of parental bedtime on children’s screen time was observed (direct effect: β = 0.027, *p* = 0.689; total effect: β = 0.068, *p* = 0.308). Parental control was found to be associated with a reduction in screen time among elementary and junior high school students.		
Authors, Year, Study Design		Age (yrs)	*N* and Statistical analysis	BMI Measure	PA Measure	Screen Time Measure	Family Environment and Characteristics	Nutritional Habits	Main Findings		
Bracale et al., 2015 [41]. Cross-sectional		6–11	*N* = 14,500 children Comparative analyses with ANOVA, multinomial regression	A questionnaire with the following information: children’s anthropometric measures	A questionnaire with the following information: physical activity frequency	A questionnaire with the following information: TV watching time	A questionnaire with the following information: parents’ and children’s ages, gender, citizenship, and the level of parents’ education	A questionnaire with the following information: parents’ and children’s fruit and vegetable consumption frequencies	Important environmental factors which contribute to childhood obesity and being overweight include screen time, physical activity, vegetable intake, and the level of parental education. While eating fruits and vegetables alone may not prevent obesity, children who eat vegetables less often are typically more sedentary and tend to watch more TV. Regular physical activity, even just 2–3 times a week, can help lower the risk of obesity. On the other hand, excessive TV watching encourages the consumption of unhealthy foods and fosters an unhealthy lifestyle, making it a significant risk factor for obesity.		
Authors, Year, Study Design		Age (yrs)	*N* and Statistical Analysis	BMI Measure		PA Measure	Family Environment and Characteristics		Main Findings		
Brouwer et al., 2018 [33]. Cross-sectional		4–7	*N* = 623 children and 606 families Descriptive statistics, non-parametric correlation (Spearman), linear regression, and stratification	Height and weight were measured by trained youth healthcare nurses at age 6 during a routine check-up. Parents’ heights and weights were self-reported via questionnaires.		PA in children was assessed using an ActiGraph GT3X (ActiGraph, Pensacola, FL, USA), and parental PA was assessed using the validated Short Questionnaire to Assess Health Enhancing Physical Activity (SQUASH).	Socioeconomic status (SES) was assessed by education and the income level of the parents and the highest household income.		The parents’ overall physical activity (PA) was not found to be correlated with the PA levels of their children (mothers: r = 0.018, *p* < 0.05; fathers: r = 0.010, *p* < 0.05). However, light PA by the fathers was linked to lower-to-moderate physical activity (MPA) levels in their children. Mothers who engaged in more vigorous physical activity (VPA) and participated in sports and leisure activities had daughters who were more active in moderate-to-vigorous physical activity (MVPA) (r = 0.159, *p* < 0.05). Maternal PA showed a significant relationship with PA levels in girls, while paternal PA levels were associated with PA in boys. More time spent performing light PA was connected with greater sedentary times and less MVPA in children.		
Authors, Year, Study Design		Age (yrs)	*N* and Statistical Analysis	BMI Measure	PA Measure	Screen Time Measure	Family Environment and Characteristics		Main Findings		
Cadogan et al., 2014 [40]. Cross-sectional		9	*N* = 8568 children Multivariate analysis, multinomial multivariate logistic regression	Height and weight data were recorded using a Leicester portable height stick and an SECA 761 flat mechanic scale.	PA questions adapted from the Leisure Time Exercise Questionnaire	Combining three screen time variables: hours spent watching TV or videos, playing video games, and using a computer	Primary caregiver’s education, employment status, parenting style, primary caregiver weight status, siblings, and household structure		A total of 26.3% of the participants showed low physical activity (PA), 19.3% had moderate levels, and 54.4% displayed high activity levels. Boys were more likely to meet the recommended PA guidelines than girls (29% versus 21%, *p* < 0.001). At the individual level, factors such as participation in sports or fitness clubs and having an active favorite hobby were strongly linked to higher PA levels in 9 year-olds. On the other hand, exceeding 2 h of screen time and being overweight or obese were associated with lower PA levels.		
Authors, Year, Study Design		Age (yrs)	*N* and Statistical Analysis		Sleep Measure		Main Findings				
Chehri et al., 2022 [34]. Cross-sectional		4.5–6	*N* = 153 children Non-parametric tests and multiple linear regression		Quality of sleep was measured with the Pittsburg Sleep Quality Index (PSQI), the Sleep Hygiene Index (SHI), and the Child Sleep Habit Questionnaire (CSHQ).		A strong and significant connection exists between the sleep habits of children in daycare and the quality of their parents’ sleep, where poor sleep quality in parents tends to lead to difficulties in their children’s sleep patterns. The quality of parents’ sleep was found to positively influence and predict changes in their children’s sleep habits. Additionally, the study emphasized that parental sleep hygiene plays a crucial role in shaping children’s sleep behaviors, with the level of parental involvement and guidance on sleep hygiene as key factors. Parents experiencing sleep disturbances were more likely to encounter challenges with their children’s sleep, indicating that children’s sleep habits are closely aligned with their parents’ sleep practices and hygiene.				
Authors, Year, Study Design		Age (yrs)	*N* and Statistical Analysis	BMI Measure		PA Measure	Family Environment and Characteristics		Main Findings		
Dozier et al., 2020 [35]. Cross-sectional		8–12	*N* = 132 children Linear regression, interaction analysis, group comparisons, and model fit assessment	Height and weight were measured following standard procedures, and child BMI was calculated using age- and sex-specific growth charts from the CDC.		Parent and child PA data were collected using a self-reported paper-and-pencil survey adapted from the reliable and valid Leisure Time Exercise Questionnaire.	Self-reported demographic measures for parents and their children were collected. The child’s perception of parental support for PA was measured with a 9 item scale adapted from reliable tools.		Overall, 33% of the children adhered to physical activity (PA) guidelines. Boys reported a higher level of perceived parental support for PA (20.22 ± 3.059) than girls (19.11 ± 3.80) and greater participation in sports. However, these differences were not statistically significant. About 57% of the parents met PA guidelines. No significant association was observed between the parents and children meeting PA guidelines in the overall sample (boys: OR = 1.43, 95% CI = 0.63–3.24, *p* = 0.39; girls: OR = 0.65, 95% CI = 0.18–2.33, *p* = 0.51). Among boys, those with parents who met PA guidelines were 3.8 times more likely to meet PA guidelines themselves (*p* = 0.04, 95% CI 1.28–13.41). Parental support for PA is linked to higher levels of physical activity in children.		
Authors, Year, Study Design		Age (yrs)	*N* and Statistical Analysis	BMI Measure	Mental Health Measure		Family Environment and Characteristics	Nutritional Habits Measure	Main Findings		
Eley et al., 2004 [53]. Cross-sectional		15–19	*N* = 1294 parents and 1.818 adolescents Logistic regression, robust cluster option, and univariate and multivariate analysis	Parental BMI was calculated as the standard weight ratio in kilograms divided by the square height in meters.	Parental characteristics were assessed using the short form of the neuroticism scale from the Eysenck Personality Questionnaire, the General Health Questionnaire, and short versions of the anxious arousal and high positive affect subscales from the Mood and Anxiety Symptoms Questionnaire. Adolescent characteristics were evaluated through self-reported depression levels using the short form of the Moods and Feelings Questionnaire (SMFQ).		Social adversity was assessed using the Social Problems Questionnaire; parental educational level was assessed using an 8 point scale ranging from no qualifications to a postgraduate degree; and adverse life events were assessed using the 12 item List of Threat-Ending Events.	Alcohol intake was assessed as the number of units consumed per week.	A nearly significant correlation of 0.046 (*p* = 0.054) was found between age and depressive symptoms. Girls exhibited significantly higher depression scores compared with boys (Gmean = 8.59, SD = 5.92; Bmean = 6.73, SD = 4.91). Parental smoking (OR = 1.26, *p* < 0.01), parental BMI (OR = 1.33, *p* < 0.001), parental education level (OR = 2.63, *p* < 0.001), and social problems (OR = 2.11, *p* < 0.001) showed significant associations with adolescent depressive symptoms. Parental education and social issues were strongly linked to more severe depressive symptoms in adolescents.		
Authors, Year, Study Design		Age (yrs)	*N* and Statistical Analysis	BMI Measure	PA Measure	Screen Time Measure	Mental Health Measure	Family Environment and Characteristics	Nutritional Habits		Main Findings
Foster et al., 2020 [43]. Cross-sectional		10–17	*N* = 14,733 children and adolescents Descriptive, bivariate, and multivariate (or multivariable) analyses and complex survey sampling techniques	Parents reported their children’s weights and heights in the NSCH. BMIs were calculated from those reported measurements.	The physical activity variable queries how many days a child spent more than 60 min per day exercising or playing a sport per week.	Television and computer or other electronic device time for each child were acquired using categorical responses ranging from none to 4 or more hours, specifically asking about weekdays only.	Mental health status was generated from the question, “In general, how is your mental or emotional health?”	Race, ethnicity, and education level were collected, along with metropolitan statistical area (MSA) residence as a binary variable (yes or no). Family-level income data were available from the public use file as a percentage of the federal poverty level (FPL).	Family food security was assessed based on four parent responses: (1) always able to afford nutritious meals; (2) able to afford enough food but not always the preferred types; (3) sometimes unable to afford sufficient food; and (4) often unable to afford enough food.		In rural areas, 19.3% of children in low-income families were overweight, and 24.8% were obese, compared with 14.6% and 18.3% in high-income families, respectively. In urban areas, 16.9% of low-income children were overweight, and 23.5% were obese, while 15.8% and 12.5% were overweight and obese in high-income families, respectively. These findings highlight the role of maternal mental health in rural areas and its connection to child weight. In contrast, positive paternal mental health is linked to lower odds of obesity, with family food security also playing a role.
Authors, Year, Study Design		Age (yrs)	*N* and Statistical Analysis	PA Measure		Mental Health Measure	Family Environment and Characteristics		Main Findings		
Kobayashi et al., 2019 [45]. Cross-sectional		12–14	*N* = 280 low-income adolescents Multiple linear regression, 2 way interaction, multiple group test, and standardized regression coefficients were estimated.	Physical activity was measured using the World Health Organization’s Global Physical Activity Questionnaire.		The parents’ total stress was measured using the abbreviated version of the Hispanic stress inventory for immigrants.	Family communication was measured using the Family Relations Scale.		Parental stress was found to have a negative effect on adolescents’ MVPA levels (r = −0.158, *p* ≤ 0.05). Positive family communication had a positive influence (r = 0.171, *p* ≤ 0.05). Stress among parents was associated with poorer family communication, which in turn was positively related to physical activity in youths. The interaction between parental stress and family communication notably impacted the adolescent MVPA (β = 0.20, t = 2.471, *p* = 0.01), with a more substantial negative impact observed in boys when family communication was low.		
Authors, Year, Study Design		Age (yrs)	*N* and Statistical Analysis	BMI Measure	PA Measure		Mental Health Measure	Family Environment and Characteristics	Main Findings		
Maher et al., 2017 [42]. Cross-sectional		8–12	*N* = 191 mothers Simple linear regression, multiple linear regression, and moderation analysis	Height and weight were measured using an electronically calibrated digital scale and professional stadiometer. These data were used to calculate the body mass indexes for mothers and CDC age- and sex-specific BMI percentiles for children.	Physical activity (PA) and sedentary behavior (SB) were measured in children via an ActiGraph GT2M or GT3X accelerometer-based activity monitor (Firmware v06.02.00; ActiGraph, Pensacola, FL, USA).		Maternal mental health: Depression Scale, State-Trait Anxiety Inventory, Cohen’s Perceived Stress Scale, Parenting Stress Scale, Financial Stress Scale, and Life Events Stress Scale. Maternal well-being: Rosenberg Self-Esteem Scale and Satisfaction with Life Scale.	Questionnaires assessing age, household type, marital status, maternal employment status, maternal education level, annual household income, and race or ethnicity for themselves and their children	There was a stronger link between maternal parenting stress and children’s physical activity (PA) in single-parent households compared with dual-multigenerational households. A less conclusive association was observed between maternal parenting stress and children’s sedentary behavior (SB) in single-parent families. Children participated in just under an hour of moderate-to-vigorous physical activity (MVPA) per valid day while spending over half of their accelerometer wear time engaged in SB. Three-fourths of the children in the sample had at least 7 h of SB each day.		
Authors, Year, Study Design		Age (yrs)	*N* and Statistical Analysis	BMI Measure		Mental Health Measure	Nutritional Habits		Main Findings		
Makinen et al., 2014 [54]. Cross-sectional		M age = 14.5	*N* = 1370 adolescents Chi-squared test and Fisher’s exact test; inferential statistical analyses; binary logistic regression; and moderation analysis (linear probability model)	School nurses measured each participant’s body weight and height, and the pupils also reported their subjective body weights and heights.		Self-esteem was measured using the Rosenberg Self-Esteem Scale (RSES).	The Eating Disorder Inventory (EDI) assessed subjective eating disorder pathology. At the same time, health- and food-related attitudes and habits were examined using questions from the Finnish School Health Promotion Study and a major Finnish twin study.		The participants exhibited good self-esteem, with no significant differences between those with excess and healthy weight (mean 29.06 ± 6.03; mean 29.70 ± 5.55). Adolescents in mid-adolescence did not show a clear association between excess weight and self-esteem. Girls were more likely to engage in unhealthy weight control behaviors. Overweight boys had lower physical activity levels and spent more time watching TV than their healthy-weight counterparts. Most adolescents rated their health as good. While 90% reported having close friends without weight-related issues, those with excess weight dated less frequently.		
Authors, Year, Study Design		Age (yrs)	*N* and Statistical Analysis	BMI Measure		Sleep Measure	Family Environment and Characteristics		Main Findings		
Martins et al., 2015 [48]. Cross-sectional		4–10	*N* = 136 children. Exploratory data analysis, descriptive statistics, and inferential tests	Subjects were weighed using a calibrated scale (100 g), and their heights were measured using a wall-mounted stadiometer (accurate to 0.5 cm).		Children Sleep Habits Questionnaire validated for the Portuguese population (CSHQ-PT)	A questionnaire with demographic, health status, and anthropometric information was applied.		No significant differences in sleep disorders (SDs) were observed based on age, gender, ethnicity, or school retention. Children from single-parent households tend to experience more sleep disturbances, especially parasomnia. These children also sleep less compared with those from two-parent families, with higher incidences of bedtime resistance and parasomnia linked to the family structure.		
Authors, Year, Study Design		Age (yrs)	*N* and Statistical Analysis	BMI Measure		Family Environment and Characteristics	Nutritional Habits		Main Findings		
Qiu et al., 2023 [14]. Cross-sectional		7–12	*N* = 242 children Descriptive statistics, linear regression, nonparametric tests, and multicollinearity check	Children’s weights and heights were obtained from annual school health records.		Parents were required to self-report their sex, age, education, and family income. Parental education was categorized as “secondary high school or lower”, “high school or equivalent”, “bachelor’s degree or equivalent”, and “master’s degree and above”.	Parental feeding practices were assessed using parental feeding behaviors and Practice Food Preferences Questionnaire (PFPQ); Children’s eating behaviors were assessed using the Children’s Eating Behavior Questionnaire. Food preferences were evaluated through a questionnaire covering 79 commonly consumed food items.		Parental feeding practices and children’s eating behaviors did not show significant differences in children aged 7–12 years (*p* > 0.050 for all). Emotional feeding by parents was positively associated with emotional undereating in children (β 0.54, 95% CI 0.16–0.92, *p* = 0.006). Instrumental feeding negatively affected the children’s preference for fish (β −0.47, 95% CI from −0.94 to −0.01, *p* = 0.048). Parental encouragement in feeding was positively associated with children’s preference for processed meat (β 0.43, 95% CI 0.08–0.77, *p* = 0.015). Parents of boys tended to use more overeating control practices compared with those with girls. Children were more likely to exhibit emotional undereating when their parents had higher emotional feeding scores.		
Authors, Year, Study Design		Age (yrs)	*N* and Statistical Analysis	BMI Measure	Mental Health Measure		Family Environment and Characteristics	Nutritional Habits	Main Findings		
Sepúlveda et al., 2020 [52]. Cross-sectional		8–12	*N* = 239 families Descriptive statistics, partial correlation, MANCOVA, post hoc tests, and mediation analysis	Height and weight were measured using a digitally calibrated scale (Type SECA 799 and 769) and a telemeter.	Psychological distress was assessed using the Trait-Anxiety Scale of the Trait-State Anxiety Inventory for Children (STAIC) and the Children Depression Inventory (CDI).		The family environment was assessed using the EE (Family Questionnaire (FQ)) and the Family Adaptation and Cohesion Scales (FACES-III).	Disordered eating symptomatology was assessed using the Body Esteem Scale (BES) and the Children’s Eating Attitudes Test (ChEAT). Trained interviewers evaluated the presence of episodes of LOC eating through a semi-structured interview about the child’s eating habits and behavior.	A child’s BMI was associated with the family’s expressed emotion (EE), the child’s psychological distress, and disordered eating behaviors. No significant indirect effect of EE on the depression levels was found ([B] = 0.07, [SE] = 0.07, 95% CI: 0.07–0.23). A significant relationship was observed between high EE and loss of control (LOC) eating episodes (B[SE] = 0.47 [0.22]; *p* = 0.03). Both EE and psychological distress were linked to LOC eating, while the BMI was related to psychological distress and disordered eating symptoms but not family functioning. Children with LOC eating patterns also had higher family EE and emotional distress levels compared with those without LOC episodes, with no significant differences in the family environment.		
Authors, Year, Study Design		Age (yrs)	*N* and Statistical Analysis	BMI Measure	PA Measure		Mental Health Measure	Family Environment and Characteristics	Nutritional Habits		Main Findings
Tommasi et al., 2022 [37]. Cross-sectional		8–11	*N* = 125 children and 161 parents Descriptive statistics, reliability analysis (Cronbach’s alpha), bivariate correlations, multigroup confirmatory factor analysis (MG-CFA), and MIMIC models	The anthropometric assessment used a meter to measure body stature and a scale to measure body weight. Parents were asked if they were overweight.	The children’s PA was measured through a short questionnaire asking if they played sports, and the parents were asked if they played sports.		Measured with two scales taken from the SCARED. The Parenting Stress Index (short form), the Child Behavior Check List/6–18, the Parent Form, and the Marlowe-Crowne–Scale were used. It was also asked if they were deemed fat by their classmates.	A short questionnaire assessed parent-child relationship satisfaction based on time spent by mothers and fathers. Personal data like gender and age were also collected from parents.	Measured with a short questionnaire. Children were asked if they liked eating. The Children’s Eating Behavior Questionnaire (CEBQ) was used. Parents were asked if they followed a diet.		Children’s eating habits were not significantly influenced by their BMIs, except in the case of mothers. There was a link between children’s eating habits and parental concern about their BMIs. Children’s anxiety did not appear to affect their eating behaviors. Both children’s eating habits and parental stress levels similarly predicted children’s mental health, with better mental health observed in both parents and children when children enjoyed their meals. Additionally, children’s strong desire to drink was associated with higher parental stress levels.
Authors, Year, Study Design		Age (yrs)	*N* and Statistical Analysis	BMI Measure		Screen Time Measure	Family Environment and Characteristics		Main Findings		
Tsai et al., 2024 [49]. Cross-sectional		6–9	*N* = 246 children Descriptive analysis, simple linear regression, multiple linear regression, and multicollinearity analysis	Children’s weights were measured using a digital scale (HD-380, Tanita), and heights were measured using a portable stadiometer (Model 213, Seca)		Children’s sleep habits were assessed using the Children’s Sleep Habits Questionnaire, Pittsburgh Sleep Quality Index, and Centre for Epidemiologic Studies questionnaires. Sleep patterns were objectively measured over seven consecutive days using a wrist-worn ActiGraph (Actiwatch 2, Phillips-Respironics Co., Inc., Murrysville, PA, USA).	Parents provided sociodemographic information. Depression scale was included.		Overall, 60.2% of the children followed bedtime routines at least five times a week, and 84.6% had a CSHQ sleep disturbance score greater than 41. Poor sleep quality in parents was linked to more severe sleep disturbances in their children in the unadjusted and adjusted models (b = 0.48, *p* = 0.001 and b = 0.46, *p* < 0.001, respectively). Both the parents and their overweight or obese school-age children experienced significant sleep disturbances (b = 0.14, *p* < 0.001). Parents also reported elevated depressive symptoms. There was a strong bidirectional association between sleep disturbances in both parents and children who were overweight or obese, with sleep problems often clustering in these children.		
Authors, Year, Study Design		Age (yrs)	*N* and Statistical Analysis	BMI Measure	PA Measure		Mental Health Measure	Family Environment and Characteristics	Nutritional Habits	Main Findings	
Zhu et al., 2024 [44]. Cross-sectional		6–12	*N* = 388 students Descriptive statistics, group comparisons (*t*-test, ANOVA, and chi square or Fisher’s exact test), correlation analysis, and multiple linear regression	Heights and body weight were self-reported by parents.	Physical activity was measured with the short Chinese version of the International Physical Activity Questionnaire (IPAQ-C).		Social anxiety was assessed using the Social Anxiety Scale for Children (SASC). Depressive symptoms were assessed using the Depression Self-rating Scale for Children (DSRSC).	The researchers designed a sociodemographic questionnaire covering children’s ages, genders, ethnicities, family structures, parents’ education, work statuses, and residences. They used the Family Environment Scale, Family APGAR, s-EMBU-c, Kansas Marital Satisfaction Scale, and Social Support Rating Scale.	Eating behavior was assessed using a validated Chinese version of the Dutch Eating Behavior Questionnaire (DEBQ-C). The home Food Environment Scale was used, and the Child Feeding Questionnaire (CFQ) was used.	Age, father’s employment status, social anxiety, maternal punishment and harshness, parental nutrition literacy, perception of child weight, pressure to eat, and family functioning play a significant role in shaping the eating behaviors of overweight or obese children. Movement behaviors do not appear to influence eating patterns. Family structure does not significantly impact children’s eating behaviors, with no notable differences found between those living with grandparents, parents, or other family arrangements. Maternal strictness or punitive styles are associated with poorer eating habits. At the same time, lower parental nutrition literacy is linked to unhealthy eating, particularly emotional eating; parents who perceive their child as overweight attempt to change their child’s eating behaviors.	
Authors, Year, Study Design		Age (yrs)	*N* and Statistical Analysis	BMI Measure		PA Measure	Family Rnvironment and Characteristics		Main Findings		
Thompson et al., 2010 [39]. Cross-sectional		10–11	*N* = 30 parents. The interviews were recorded using an Olympus DS-2200 Digital recorder and a semi-structured script with follow-up probes on key topics of interest.	Telephone interviews		Telephone interviews	Telephone interviews		Parents consider family involvement in physical activity necessary but feel the specific activities are less crucial. While they recognize the benefits of exercise, families often fail to engage in physical activities during the week. Weekends are typically filled with sedentary activities like eating, watching TV, gardening, or playing games. Key obstacles to family physical activity include children’s varying ages and interests and hectic schedules.		
Authors, Year, Study Design		Age (yrs)	*N* and Statistical Analysis		Mental Health Measure		Family Environment and Characteristics	Main Findings			
Benny et al., 2023 [55]. Longitudinal study: wave 1 = 2016–2017; wave 2 = 2017–2018; wave 3 = 2018–2019		M age = 14.9 ± 1.0	*N* = 21,141 adolescents Three-level multilevel modeling (MLM); intraclass correlation coefficient (ICC); bivariate multilevel analyses; and adjusted multilevel model		Adolescent depression and anxiety: assessed using self-reporting measures with the 10 item Center for Epidemiologic Studies Depression Scale-Revised and the 7 item Generalized Anxiety Disorder scale (GAD-7)		Income inequality: measured using the Gini coefficient (CDs). Students reported age, gender, race, and personal weekly spending money.	The mean Gini coefficient was 0.37 (range: 0.30–0.46). Attending schools in communities with higher income inequality was associated with increased depressive scores (β = 0.08; 95% CI = 0.02, 0.14) and greater odds of depression (odds ratio = 1.55; 95% CI = 1.06, 2.28). Income inequality did not significantly relate to anxiety symptoms over time. It was linked to higher depressive scores in both females (β = 0.10; 95% CI = 0.01, 0.18) and males (β = 0.08; 95% CI = 0.01, 0.15), as well as increased anxiety scores in females (β = 0.13; 95% CI = 0.04, 0.22) but not in males (β = 0.01; 95% CI = −0.09, 0.06).			
Authors, Year, Study Design		Age (yrs)	*N* and Statistical Analysis	BMI Measure	PA Measure		Mental Health Measure	Family Environment and Characteristics	Nutritional Habits		Main Findings
Harbec et al., 2017 [46]. Observational, longitudinal cohort study: 2 data collection points (age 6 and 10)		6–10	*N* = 1492 children Ordinary least-squares (OLS) regression	Maternal body mass index (BMI) and child BMI were directly measured in children aged 1.5 and 6 years.	Parents reported on child’s physical fitness relative to other children (ranging from 5 (much more than others) to 15 (much less than others)).		Children self-reported their social adjustment using the Social Behavior Questionnaire. Mothers reported their depressed feelings at 5 months using a short version of the Center for Epidemiological Studies Depression Scale.	Mothers reported education (0 = completed high school, 1 = not completed), family structure (0 = intact, 1 = not intact), and family dysfunction using a 7 item scale measuring communication, problem-solving, behavior control, and affection.	The scale measuring family meal environment quality, as reported by parents, assessed factors like enjoyment, conversation, acceptance, and openness. Parents also reported their frequency of soft drink consumption, with responses ranging from 1 (never) to 7 (4 or more times per day).		A positive family meal environment at age 6 is linked to healthier lifestyle habits at age 10 (B = −0.01, *p* = 0.014, 95% CI: from −0.01 to −0.002). Higher maternal depression levels were associated with a lower quality of family meal environment (B = −0.01, *p* = 0.003, 95% CI: from −0.02 to −0.004). Improved family meal quality led to better physical fitness and reduced soft drink consumption.
Authors, Year, Study Design		Age (yrs)	*N* and Statistical Analysis	BMI Measure	PA Measure		Screen Time Measure	Family Environment and Characteristics	Main Findings		
Hoehne et al. 2024 [36]. Longitudinal study: T1 and T2 (M = 273 days, SD = 55 days)		6–11	*N* = 971 children Multilevel modeling (hierarchical linear modeling)	Measured using calibrated ultrasound measurement devices and calibrated digital scales.	Leisure time activity measured: daily hours of physical activity. Parents reported their children’s time each day in three different activities. Questions were answered on a 5 point Likert-type scale (0 = “no time at all”, 1 = “approximately 30 min”, 2 = “1–2 h”, 3 = “3–4 h”, 4 = “more than 4 h”).		Leisure time activities measured: (1) daily hours of watching TV and (2) daily hours of using computers. Parents reported their children’s time each day in three different activities. Questions were answered on a 5 point Likert-type scale as used in PA measurement.	Parental education level was used as a measure of socioeconomic status on a scale from 1 (“none”) to 6 (“university degree”). Parents’ highest job level was used as a second indicator of socioeconomic status on a scale from 0 (lowest job level) to 4 (highest job level).	Parental education and job status were associated with children’s BMIs, with lower education and job statuses linked to higher average BMIs. Children with more daily physical activity had lower BMIs than those who spent more time watching TV. The prevalence of being overweight and obese was notably higher among children with less educated or lower-status parents. In a longitudinal study, boys experienced an average BMI increase of 0.58 per year, while girls saw an increase of 0.18. A correlation (r = 0.35) indicated that children starting with a higher BMI tended to increase over time. Higher parental education and job statuses were predictors of lower cross-sectional BMIs but did not impact longitudinal BMI changes. Increased consumption correlated with a more significant rise in BMI, while more physical activity predicted a decrease in the BMI slope. Changes in TV viewing hours and physical activity impacted the BMI in opposite directions.		
Authors, Year, Study Design		Age (yrs)	*N* and Statistical Analysis	BMI Measure	Mental Health Measure		Family Environment and Characteristics	Nutritional Habits	Main Findings		
Monteiro et al., 2004 [51]. Observational, longitudinal cohort study: 2 data collection points (1997–1998, sample loss: 4.7%)		15–16	*N* = 1076 adolescents Linear regression (bivariate analyses and multivariate analyses)	UNICEF portable electronic scales (Uniscale) and height without shoes	Behavioral and psychological questionnaire		Socioeconomic and demographic questionnaire	Dietary questionnaire	Obesity occurrence did not differ by skin color, but sex showed a borderline significant relationship (*p* = 0.062). A positive association was found between obesity and household income. Children who watched TV for 4 h or less had half the risk of obesity compared with those watching for 5 or more hours. Boys with anxiety traits had a fourfold higher obesity risk. Smoking and fat intake were not associated with obesity.		
Authors, Year, Study Design		Age (yrs)	*N* and Statistical Analysis	BMI Measure	Screen Time Measure		Family Environment and Characteristics	Nutritional Habits Measure			Main Findings
Neumark-Sztainer et al., 2015 [50]. Longitudinal study: 2 data collection points (1998/1999 and 2003/2004)		μ = 14.4 + 1.7	*N* = 314 pairs of parents and overweight adolescents Logistic regression; interaction analysis; propensity score weighting	Adolescents’ heights and weights were measured, and self-reported data were collected. Parents were asked to describe their child’s current weight using six categories: “very underweight”, “somewhat underweight”, “about right”, “somewhat overweight”, “very overweight”, and “don’t know”. Parents also self-reported their heights and weights.	To assess television-watching during meals, parents were asked how strongly they agreed with the statement, “We often watch TV while eating dinner”.		Adolescent gender, age, and ethnicity or race were based on adolescent self-reporting. Parental gender and family socioeconomic status (SES) were based on parental responses. SES was based on a factor analysis using parental education of the more highly educated parent, family income, and the higher employment status of the father or mother.	Family meal frequency was measured using the question, “How many times did your family eat a meal together in the past week?” Fast food consumption was assessed with the question, “How many times was a family meal from a fast-food restaurant (including pizza) eaten at home or the restaurant in the past week?” Fruit and vegetable availability at home was evaluated by asking parents, “How often are fruits and vegetables available in your home?”			No significant differences were found in family meal practices, the availability of healthy or unhealthy foods at home, or parental encouragement for adolescents to make healthy food choices and stay active. Parents who correctly identified their children as overweight were more likely to encourage dieting for weight control. Encouragement to diet at time 1 was marginally linked to a persistent overweight status in boys (OR: 3.54 [CI: 0.90–13.85]; *p* = 0.070) and significantly for girls (OR: 2.98 [CI: 1.10–8.07]; *p* = 0.032). About half of the boys whose parents did not encourage dieting remained overweight, while a similar pattern was observed for girls.
Authors, Year, Study Design	Age (yrs)		*N* and Statistical Analysis	BMI Measure	Mental Health Measure		Family Environment and Characteristics	Nutritional Habits Measure	Main Findings		
Peng L, et al., 2023 [56]. Longitudinal study- data 1 (2020) and data 2 (2021)	13–15		*N* = 7645 students Descriptive statistics, chi-squared test or *t*-test, multiple logistic regression, generalized estimation model (GEM), sided tests	BMI was calculated concerning the Chinese children’s standard.	Parental anxiety variables were measured using the Anxiety Self-Assessment Scale (SAS).		A basic information questionnaire (gender, grade, and age of the elementary school students, as well as education level, age, and residence) was used to collect demographics about the subjects and their parents. Family environment variables were measured using the Chinese Family Assessment Instrument (C-FAI).	Children’s nutritional statuses were assessed using BMIs based on the Chinese standard for children. Since June 2020, parents have been asked about changes in meal patterns and their consumption of major food items during the pandemic. They were explicitly asked whether their household consumption of “staple foods”, “vegetables”, “fruits”, and “sugary drinks and desserts” has “increased”, “remained the same”, or “decreased”.	Wave 1: The prevalence rates were 11.64% for malnutrition, 13.11% for being overweight, and 11.60% for obesity. Wave 2: Malnutrition prevalence decreased to 4.96%, overweight status increased to 13.73%, and obesity dropped to 10.60%. Significant differences in malnutrition and obesity rates between the waves were observed (*p* < 0.05). Parental anxiety was more common in Wave 1, and environmental barriers were higher for malnourished children during this period. Wave 2: Families reported decreases in staple food, vegetable, and fruit consumption of 22.26%, 33.23%, and 32.28%, respectively, while 50.70% increased their intake of sugary beverages and desserts. Wave 2: Child malnutrition was more prevalent in households with reduced staple food intake (OR = 1.88, 95% CI: 1.05–3.37). Increased take-out and meal delivery correlated with higher overweight rates (OR = 1.33, 95% CI: 1.02–1.75), and increased sugary drink consumption was linked to higher obesity rates. Risk factors for obesity included low maternal education, younger ages, urban living, family dysfunction, and parental anxiety (*p* < 0.05).		
Authors, Year, Study Design		Age (yrs)	*N* and Statistical Analysis	BMI Measure	PA Measure	Mental Health Measure	Family Environment and Characteristics	Nutritional Habits			Main Findings
Wang et al., 2023 [47]. Longitudinal cohort study: data were collected across 27 years for NHSII (1989–2016) and 20 years for GUTS (1996–2016), with biennial follow-ups every 1–3 years.		9–17	*N* = 13,478 children and 10,368 mothers Generalized estimating equations; multiple imputation; mediation analysis	Body height and weight were asked for in biennial questionnaires.	Mothers’ physical activity was assessed by a validated, self-reported questionnaire.	Studies Depression Scale-10 (CESD-10) results were collected.	Smoking was asked about in biennial questionnaires.	A food frequency questionnaire (FFQ) was distributed. Questions about the consumption of alcoholic beverages were included in each questionnaire. Diet quality was calculated using the Alternate Healthy Eating Index 2010 (AHEI) diet score.			Higher maternal healthy lifestyle scores were associated with lower depression levels in children. Children of mothers with the healthiest lifestyle had depression scores 0.30 lower (95% CI: 0.09–0.50) compared with those with the least healthy lifestyles. Children of mothers with normal BMIs had lower depression scores than those with overweight or obese mothers. Mothers who engaged in regular physical activity (≥150 min/week) had children with lower depression scores. Maternal healthy lifestyle scores were linked to higher healthy lifestyle scores in children, with those of mothers with the highest scores showing better health and fewer depressive symptoms.
Authors, Year, Study Design	Age (yrs)		*N* and Statistical Analysis	BMI Measure		PA Measure		Nutritional Habits	Main Findings		
Webber-Ritchey KJ, 2023 [6]. Descriptive longitudinal study with data from a CHECC (2015–2019)	6–12		*N* = 428 dyads Descriptive statistics, regression models (linear and logistic), and factor analysis	The height and weight of each parent and child were collected and then used to compute a BMI z-score.		Children were surveyed about the typical number of hours they spend on sedentary activities each day during weekdays and weekends. They were questioned about their participation in intense exercise for at least 20 min and moderate exercise for at least 30 min over the past 7 days. Parents were asked how many days per week they typically engage in activities with their children.		The parents’ consumption of 24 common healthy and unhealthy foods in the prior month was assessed using adapted questions from Supplemental Nutrition Assistance Program Education (SNAP-Ed).	A total of 223 children (52.10%) were classified as having normal weights, 73 children (17.06%) were considered overweight, and 132 children (30.84%) were considered obese. Among parents, 56 (13.08%) were of a normal weight, 122 (28.5%) were overweight, and 250 (58.41%) were obese. Time spent on the internet was significantly associated with an increase in children’s BMIs. No correlation was identified between children’s physical activity (PA) levels and BMIs. The study highlighted that parents who frequently consumed cheese and Mexican-style salsa with tomato or tomato-based sauces exhibited significantly higher odds of being overweight or obese. Parents who consumed milk and potatoes daily had lower odds of being overweight or obese. The study identified a link between the consumption of obesogenic foods and an increased risk of obesity in children. The risk of children being overweight or obese increased by 12% if their parents were overweight or obese.		

## Data Availability

Data are contained within the article and Supplementary Materials.

## Supplementary Materials

The following supporting information can be downloaded at: https://www.mdpi.com/article/10.3390/children12020203/s1, Table S1: PRISMA 2020 Checklist. Reference [107] is cited in the Supplementary Materials.
